# Does Ageism Widen the Digital Divide? And Does It Vary by Gender?

**DOI:** 10.1093/geroni/igaa057.1007

**Published:** 2020-12-16

**Authors:** Eun Young Choi, Youngsun Kim, Edson Chipalo, Hee Yun Lee

**Affiliations:** 1 Leonard Davis School of Gerontology, Los Angeles, California, United States; 2 Kyunghee University, Yongin-si, Republic of Korea; 3 University of Alabama, Tuscaloosa, United States; 4 University of Alabama, Tuscaloosa, Alabama, United States

## Abstract

Existing literature informed that ageism might affect Internet use among older adults, further widening the digital divide among age groups. However, little empirical studies have been conducted on this topic. Our study aims to investigate (1) the current use of the Internet by gender, (2) the association between ageism and Internet use, and (3) potential gender differences. A cross-sectional data drawn from the 2016 Health and Retirement Study (HRS) was analyzed. Separate multiple regression analyses were conducted by gender to determine the varying impact of ageism on Internet use. We used two types of ageism (1) internal ageism (ingroup discrimination) and (2) external ageism (discrimination from external entities) to observe each type’s contribution to Internet use. About half of the sample (52% male and 54% female) reported using the Internet “daily,” while a quarter (26% male and 25% female) responded, “never/not relevant.” No significant differences between gender were found in levels of Internet use, the rates of external ageism, or the degree of internal ageism. A higher level of ageism was associated with a lower level of Internet use. Interaction effects between age groups and ageism varied across gender: external ageism had interaction effects on men’s Internet use whereas internal ageism showed significant results for women. Our findings suggest that ageism may influence Internet use and its impact differs by gender. Gender-tailored intervention strategies should be developed to help older individuals to diminish the adverse effects of ageism on Internet use.

